# A New Endolysin Lys59: A Broad-Spectrum Phage Endolysin Targeting Both Gram-Negative and Gram-Positive Bacteria

**DOI:** 10.3390/microorganisms14051027

**Published:** 2026-04-30

**Authors:** Yunhan Zhang, Chenwei Deng, Yanni Liu, Weiqing Lan, Yong Zhao, Xiaohong Sun

**Affiliations:** 1 College of Food Science and Technology, Shanghai Ocean University, Shanghai 201306, Chinawqlan@shou.edu.cn (W.L.);; 2 Shanghai Aquatic Products Processing and Storage Engineering Technology Research Center, Shanghai 201306, China; 3 Laboratory of Quality & Safety Risk Assessment for Aquatic Products on Storage and Preservation (Shanghai), Ministry of Agriculture, Shanghai 201306, China

**Keywords:** *Klebsiella pneumoniae*, endolysin, antimicrobial activity, biofilm

## Abstract

To address the emerging multidrug-resistance crisis caused by *Klebsiella pneumoniae*, we expressed the endolysin Lys59 derived from phage VB_KpP_HS106 and performed a comprehensive analysis of its antibacterial activity and structural features. Molecular modeling revealed that Lys59 carries a highly positively charged N-terminus and an amphipathic helix at the C-terminus. In vitro antibacterial assays showed that Lys59 exhibited significant bactericidal activity against *K. pneumoniae* with an approximately 4 log reduction at 50 µg/mL in 2 h. Meanwhile, Lys59 exhibited potent, broad-spectrum activity against both Gram-negative and Gram-positive bacteria. Stability analysis indicated that Lys59 retained high activity over a pH range of 3–9 and a temperature range of 4–55 °C. Notably, the antibacterial activity of Lys59 was found to be regulated by metal ions. Molecular docking indicated that K^+^ can enhance binding stability by interacting with ASN35 and VAL57. In contrast, Mg^2+^ and Ca^2+^ suppressed catalytic function by binding to the essential GLU17 residue. Furthermore, treatment with 200 µg/mL of Lys59 resulted in a 44.6% reduction in *K. pneumoniae* biofilm biomass. Overall, this study identified a phage-derived endolysin with broad-spectrum antimicrobial activity and demonstrated its potential as an antibacterial agent against multidrug-resistant *K. pneumoniae*.

## 1. Introduction

*Klebsiella pneumoniae*, a Gram-negative bacterium of the *Enterobacteriaceae* family, is commonly found in animals and natural environments. As a clinically important pathogen, it can cause a range of infections, including septicemia and pneumonia [1]. *K. pneumoniae* is a primary pathogen causing mastitis in livestock, resulting in substantial economic losses to the global dairy industry, with losses of approximately $1 billion annually in the United States alone [2]. The hypervirulence of *K. pneumoniae* is closely associated with factors such as its polysaccharide capsule and siderophores, while its strong biofilm-forming ability also significantly enhances drug resistance [3,4]. The World Health Organization (WHO) has included *K. pneumoniae*, specifically the emerging broad-spectrum beta-lactamase-producing and carbapenem-resistant (CRKP) strains, in its list of “priority pathogens,” recognizing it as the third-deadliest pathogen globally [5]. Therefore, the development of novel antimicrobial alternatives to control drug-resistant *K. pneumoniae* infections is urgently needed.

The potential of phage-derived endolysins as a novel therapeutic alternative to combat pathogenic bacterial infections is increasingly recognized. At present, these endolysins have been extensively applied in animal models, including mice and *Galleria mellonella*, as well as in food models [6]. Endolysin disrupts the integrity of the bacterial cell wall by hydrolyzing peptidoglycan (PG), thereby causing bacterial lysis [7]. Currently, most endolysins targeting *K. pneumoniae* are derived from phages of other genera, while endolysins derived from *K. pneumoniae* phages themselves exhibit low bactericidal activity [6]. On the other hand, the outer membrane of Gram-negative bacteria acts as a physical barrier, preventing endolysins from reaching the peptidoglycan layer, thereby limiting their therapeutic application [6]. Interestingly, some endolysins exhibit intrinsic antibacterial activity against Gram-negative bacteria. Wang et al. [8] reported that the endolysins (Lysin1 and Lysin2) reduced the viable count of *S. enteritidis* 35 by approximately 1 log within 30 min. These endolysins ordinarily possess a cationic or amphipathic region at the C-terminal that enables penetration of the outer membrane (OM) by interacting with negatively charged surface lipopolysaccharide (LPS). Therefore, researchers have adopted two main strategies: on the one hand, screening for endolysins that possess an amphipathic helix or positive charges at the C-terminus or N-terminus to obtain candidate molecules with intrinsic antibacterial activity; on the other hand, enhancing the antibacterial activity of endolysins by combining them with outer membrane permeabilizers or fusing them with cationic peptides [7].

It is worth mentioning that temperature, pH, and metal ions can significantly affect the antibacterial activity of endolysins. Furthermore, the action of endolysins under physiological conditions remains to be further investigated. Srinivasan et al. [9] reported that the activity of Vplys60 is significantly influenced by pH, temperature, and metal ions. It remained stable within a pH range of 6–10 and at temperatures from 37 to 75 °C. Notably, Ca^2+^ enhanced its activity, whereas other metal ions exerted inhibitory effects. Additionally, the endolysin LysSE24 contains the Lyz endolysin autolytic domain and exhibits high lytic activity against *Salmonella* [10]. Therefore, studying these endolysins with inherent antibacterial activity may provide new insights for identifying novel endolysins with potent antibacterial efficacy or optimizing the modification of natural endolysins.

This study aims to express an endolysin with broad-spectrum antibacterial activity. Based on this, the structural features and antibacterial activity of the endolysin were investigated, and the effects of temperature, pH, and metal ions on its antibacterial activity were further examined.

## 2. Materials and Methods

### 2.1. Strains, Plasmids and Phages

*K. pneumoniae* strains listed in Table 1 *S. aureus* were cultivated in Mueller–Hinton broth (MHB). The other strains were grown in Luria–Bertani (LB) agar and broth. All cultures were incubated at 37 °C. *K. pneumoniae* phage VB_KpP_HS106 was isolated from a sewage sample collected at the Shanghai Sixth People’s Hospital [11]. The complete genome sequence of this phage has been submitted to the GenBank database and assigned the accession number OP764672.1. For recombinant plasmid construction and expression, *E. coli* DH5α and *E. coli* BL21(DE3) were utilized.

### 2.2. Bioinformatic Analysis of the Endolysin Lys59

The endolysin Lys59 sequence was obtained through NCBI BLAST (version 2.16.0+) (BLAST: Basic Local Alignment Search Tool) analysis of the complete genome of phage VB_KpP_HS106. Phylogenetic tree construction was performed using MEGA11.0 software. Multiple sequence alignment was conducted using Clustal W, and the phylogenetic tree was inferred using the Neighbor-joining method with 1000 bootstrap replicates. The amino acid identity heatmap was generated using Bioinfo Intelligent Cloud (BIC—Bioinfo Intelligent Cloud). The domain analysis was performed using NCBI CD-Search (https://www.ncbi.nlm.nih.gov/Structure/cdd/wrpsb.cgi, accessed on 27 April 2026) and InterPro (https://www.ebi.ac.uk/interpro/, accessed on 27 April 2026). The physicochemical properties of Lys59 were evaluated using the ProtParam and ProtScale tools (https://web.expasy.org/protscale/, accessed on 27 April 2026). The secondary structure of Lys59 was predicted using SOPMA online tool (https://npsa.lyon.inserm.fr/cgi-bin/npsa_automat.pl?page=/NPSA/npsa_sopma.html, accessed on 27 April 2026). The amphipathic helix at the C-terminus of Lys59 was predicted using NetSurfP-3.0. The electrostatic potential of Lys59 was analyzed using PyMOL3.1 to observe its charge distribution at the N-terminal and C-terminal regions. All results were visualized using VMD 1.9.3 and PyMOL.

### 2.3. Cloning, Expression, and Purification of Lys59

The gene sequence encoding Lys59 was amplified by PCR using the genomic DNA of phage VB_KpP_HS106 as the template, with primers containing restriction sites for *Eco*R I and *Xho* I (Supplementary Table S1). The amplified gene fragment was cloned into the pET-28a plasmid with a C-terminal His tag, and then transformed into *E. coli* BL21(DE3). Positive colonies were selected on LB agar plates containing kanamycin (50 µg/mL). These colonies were cultured in LB liquid medium supplemented with 50 µg/mL kanamycin until the OD600 reached 0.6. After induced with 0.5 mM IPTG and incubated at 16 °C for 16 h, the cells were centrifuged and resuspended in lysis buffer (20 mM Tris–HCl, 1 mM PMSF), followed by ultrasonication and centrifugation for supernatant collection. Recombinant protein was purified using the Coolaber His tag Protein Purification Kit, followed by desalting and buffer exchange via a pre packed desalting column, and purity was assessed by SDS-PAGE.

### 2.4. Antimicrobial Activity of Endolysin Lys59

The lytic activity of recombinant endolysin Lys59 against *K. pneumoniae* 61 was evaluated using a colony forming unit (CFU) reduction assay [12]. Briefly, bacterial cultures were grown at 37 °C to an OD_600_ of 0.6, the cell pellet was washed with 20 mM Tris-HCl buffer and then resuspended to a final concentration of 10^6^ CFU/mL. Equal volumes (100 µL) of the bacterial suspension and Lys59 solution were mixed to obtain final concentrations of 5, 20, 35, 50, 75, 100, and 150 µg/mL. After 2 h of treatment, cells were plated for enumeration.

### 2.5. Time-Killing Curve

The time-killing curve assay was performed as described with some modifications [13]. One hundred microliter of *K. pneumoniae* 61 (10^6^ CFU/mL) was mixed with 100 µL of Lys59 at a final concentration of 150 µg/mL, and Tri-HCl was used as control. The groups were incubated at 37 °C for 5, 15, 30, 60, 90, and 120 min, then diluted and plated on LB agar plates to determine the viable cell count.

### 2.6. Lytic Spectrum of Lys59

All strains listed in Table 1 were selected for evaluating the lytic activity of Lys59. The strain was grown to a density of 10^6^ CFU/mL prior to washing with 20 mM Tris-HCl buffer. Then, 100 µL of the bacterial suspension was mixed with an equal volume of Lys59 solution, achieving a final concentration of 50 µg/mL. Tris-HCl buffer was used as a negative control. Following a 2 h incubation at 37 °C, serial dilutions of the samples were prepared and plated onto LB agar. After further incubation at the same temperature, colonies were enumerated as colony forming units (CFU/mL) [14].

### 2.7. Stability Analysis of Endolysin Lys59

Thermal stability of the endolysin Lys59 was evaluated by incubating the protein for 30 min across a temperature range of 4 °C to 75 °C. One hundred microliter of Lys59 (100 µg/mL) was mixed with 100 µL of the bacterial suspension (10^6^ CFU/mL) and incubated at 37 °C for 2 h. Lys59 without thermal treatment was used as the control. To determine the effect of pH on Lys59 activity, the protein was added to a suspension of *K. pneumoniae* 61 in 20 mM Tris-HCl buffers ranging from pH 3 to 12, and 20 mM Tris-HCl buffer without the protein served as the negative control. followed by incubation for 2 h. The effect of NaCl on the lytic activity of Lys59 was determined at concentrations ranging from 0 to 100 mM [15].

The effect of metal ions was assessed according to the method described by Srinivasan et al. with minor modifications [9]. Lys59 (50 µg/mL) was incubated with bacterial suspensions (10^6^ CFU/mL) supplemented with MgCl_2_, BaCl_2_, CaCl_2_, or KCl at final concentrations of 1 mM at 37 °C for 2 h. Following incubation, samples were serially diluted and plated on LB agar to quantify viable cells. Endolysin without metal ion treatment served as the control.

### 2.8. Molecular Modeling and Docking Analysis

The three-dimensional structure of Lys59 was obtained via homology modeling using SWISS-MODEL [16]. After Ramachandran validation, it was visualized using VMD. The docking of metal ions with endolysin Lys59 was performed using AlphaFold3 [17]. Ligand information can be retrieved from the PubChem database, such as Ca^2+^ (Compound CID: 271), Mg^2+^ (Compound CID: 888), and K^+^ (Compound CID: 813). The docking results were evaluated using the ipTM and pTM scores (ipTM ≥ 0.8 and pTM > 0.5). The docking of Lys59 and peptidoglycan (Compound CID: 46173163) was performed using the HADDOCK 2.4 server [18]. The structure of Lys59 bound to metal ions, together with peptidoglycan, was uploaded and subjected to docking. Active site residues selected for docking: 17, 71, 72, 73, 76, 79, 80, 101, 104, 125, 126, 127, 128, 135. Docking results were evaluated by the average docking score (<0) and visualized using PyMOL.

### 2.9. Biofilm Elimination Assay

Biofilm elimination was assessed using the crystal violet staining method [19]. *K. pneumoniae* 61 (10^9^ CFU/mL) was inoculated at a 2% volume into a 96-well plate containing 200 µL LB broth and incubated at 37 °C for 48 h to form biofilms. After incubation, bacterial supernatants were removed, and the wells were gently washed with PBS to eliminate planktonic cells. Add 200 µL of Lys59 at different concentrations (0, 25, 50, 100, and 200 µg/mL), then incubate at 37 °C for 2 h. Next, the wells were washed gently with PBS and dried. The samples were stained with 0.1% crystal violet for 30 min. Finally, the samples were dissolved in glacial acetic acid and the absorbance was measured at 595 nm. To assess the effect of Lys59 on biofilm formation, 200 µL of LB broth containing different concentrations of Lys59 were added to a 96-well plate, followed by inoculation with 2% *K. pneumoniae* (OD_600_ = 0.6). The plate was then incubated at 37 °C for 48 h. Biofilm formation was subsequently evaluated using the same crystal violet staining method as described above, and the absorbance was measured.

### 2.10. Observation of K. pneumoniae by Scanning Electron Microscopy

The effect of Lys59 on *K. pneumoniae* 61 was evaluated via SEM following the method of Song et al. [20]. A 200 µL suspension of *K. pneumoniae* 61 at 10^8^ CFU/mL was combined with an equal volume of Lys59, yielding a final protein concentration of 50 µg/mL, and then incubated at 37 °C for 2 h. Tris-HCl buffer alone served as the negative control. After treatment, the samples were fixed with 2.5% glutaraldehyde overnight, followed by centrifugation and washing three times with PBS. The cells were then dehydrated using a gradient series of ethanol (30%, 50%, 70%, 90%, and 100%) and gold-coated under vacuum. Finally, bacterial morphology was observed using a Hitachi SU5000 scanning electron microscope.(Hitachi High-Tech, located in Shanghai, China).

### 2.11. Statistical Analyses

All experiments in this study were repeated three times and data were expressed as means ± standard deviation (SD) and differences were compared by two-way ANOVA using GraphPad Prism 9.0. Differences were considered statistically significant at *p* < 0.05.

## 3. Results

### 3.1. Expression, Purification, and Physicochemical Characterization of Lys59

**Figure 1:**
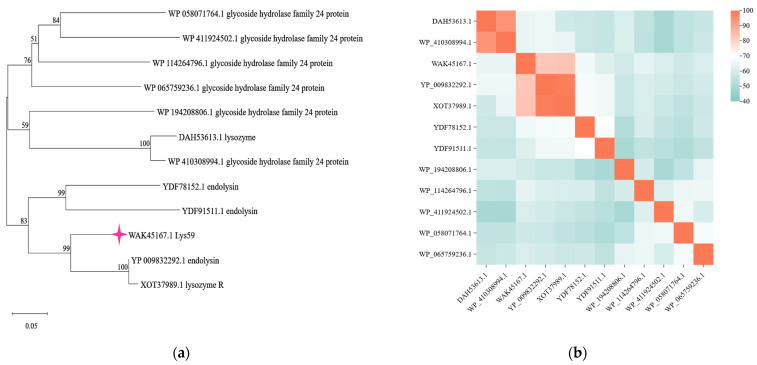
Identification of a novel endolysin Lys59 from *Klebsiella pneumoniae* phage. (**a**) Phylogenetic analysis based on NCBI BLAST alignment. (**b**) Multiple sequence alignment of Lys59 and reference sequences using Clustal Omega, with a heat map generated based on the alignment results. The color gradient represents the degree of sequence identity at each position.

The endolysin is encoded by an open reading frame (ORF59, annotated as lysozyme) in the genome of phage VB_KpP_HS106 and is named Lys59 (GenBank accession number: WAK45167.1). BLAST analysis revealed that Lys59 shares high similarity with glycoside hydrolase family 24 proteins, which are commonly associated with endolysins (Figure 1a). Clustal Omega (version 1.2.4) multiple sequence alignment showed that Lys59 shares 83.97% sequence identity with lysozyme R from *Escherichia* phage EK8 (XOT37989.1) and 83.33% sequence identity with the endolysin from *Pectobacterium* phage vB_PatP_CB1 (YP_009832292.1), respectively (Figure 1b).

**Figure 2:**
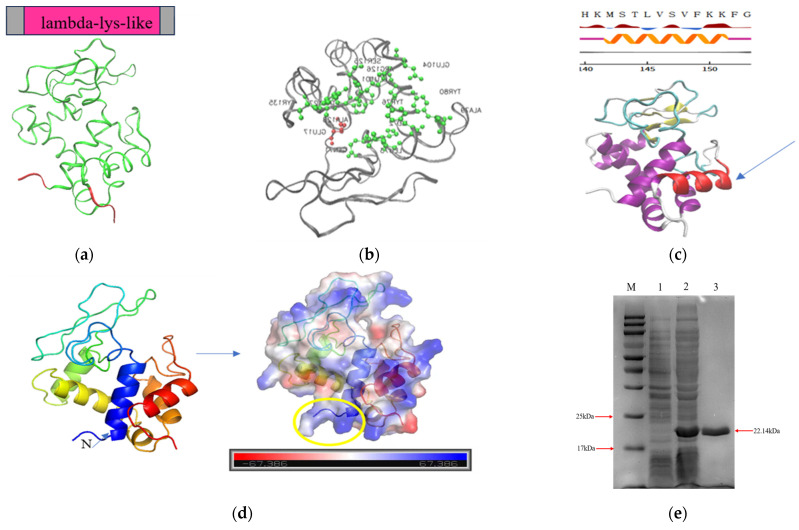
Structural feature analysis of Lys59: (**a**) Domain prediction of Lys59. (**b**) Prediction of active sites. (Red indicates catalytic residues, and green represents sugar-binding sites.) (**c**) A predicted amphipathic helix shown between amino acids 141 and 151 in Lys59. (**d**) Electrostatic surface potential of Lys59. (Positive charge is shown in blue and negative charge in red. Positive charge in the N-terminus is marked by a yellow circle.) (**e**) SDS–PAGE analysis of the purified endolysin Lys59. The theoretical molecular weight of Lys59 was approximately 22.14 kDa. M, protein molecular weight marker; 1, sonicated lysate of BL21 cells harboring the empty vector; 2, sonicated lysate of the Lys59 recombinant strain; 3, purified Lys59 protein.

Lys59 contains a lambda_lys-like domain (amino acids 4–154) (Figure 2a). Its catalytic residue is a glutamic acid (GLU) at position 17, while the sugar binding sites are located at positions 71, 72, 73, 76, 79, 80, 101, 104, 125, 126, 127, 128, and 135, corresponding to the residues Q, I, L, Y, A, Y, L, E, S, R, W, A, and Y, respectively (Figure 2b). Prot Scale analysis shows that the Grand Average of Hydropathicity (GRAVY) of Lys59 is −0.192, indicating it is a hydrophilic protein (Supplementary Figure S1 and Supplementary Table S2). The secondary structure composition of Lys59 is primarily composed of α-helix and random coil (Supplementary Figure S2). It harbors an amphipathic helix at its C-terminus (amino acids: MLVVF (hydrophobic)/STSKK (hydrophilic) (Figure 2c and Supplementary Figure S3) and carries a positively charge in the N-terminus (Figure 2d), which is a common feature of endolysins exhibiting intrinsic antibacterial activity against Gram-negative pathogens. Expression of Lys59 was carried out in *E. coli* BL21(DE3), followed by purification using nickel affinity chromatography. SDS-PAGE analysis revealed a distinct band at approximately 22 kDa (Figure 2e).

### 3.2. In Vitro Bactericidal Activity of Lys59

**Figure 3:**
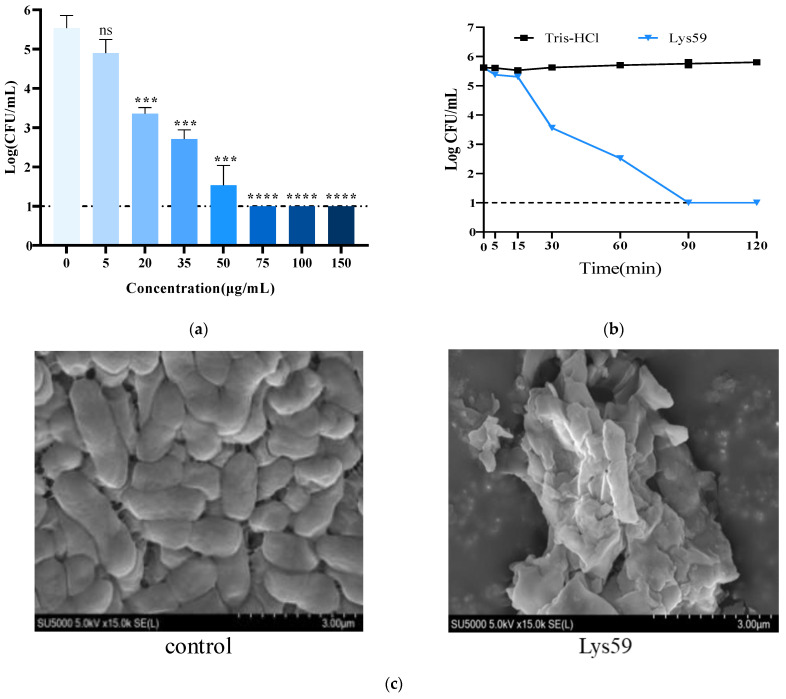
In vitro antibacterial efficacy of Lys59: (**a**) Bactericidal activity of Lys59 against *K. pneumoniae* 61. (**b**) The bacterial killing kinetics of Lys59. (**c**) Scanning electron microscopy observation of the morphological changes in bacteria after treatment with Lys59. The data are presented as the means ± standard deviations of three replicates. Statistical significance was calculated using two-way ANOVA followed by Tukey’s multiple comparison test. *** *p* < 0.001; **** *p* < 0.0001; ns, not significant.

The bactericidal activity of purified Lys59 against *K. pneumoniae* was determined by using the plate count method. Lys59 at concentrations as low as 20 µg/mL can effectively lyse bacterial cells (Figure 3a). At a concentration of 75 µg/mL, Lys59 reduced the viable cell count of *K. pneumoniae* from approximately 5.5 log CFU/mL to the undetectable level. The kinetic of bacteria killing of the Lys59 was also assessed. The Lys59 reduced the viable count of *K. pneumoniae* by 2.07 log within 30 min and decreased to undetectable level within 90 min (Figure 3b). The morphological changes in *K. pneumoniae* in response to Lys59 treatment were observed by scanning electron microscopy (SEM). The bacterial cells presented evident membrane deformation and surface collapse. The cell wall was damaged, and a large amount of cytosol leakage was observed. In contrast, the control cells remained intact with a smooth surface (Figure 3c).

### 3.3. Killing Activity of Lys59 Against Gram-Positive and Gram-Negative Bacteria

**Figure 4:**
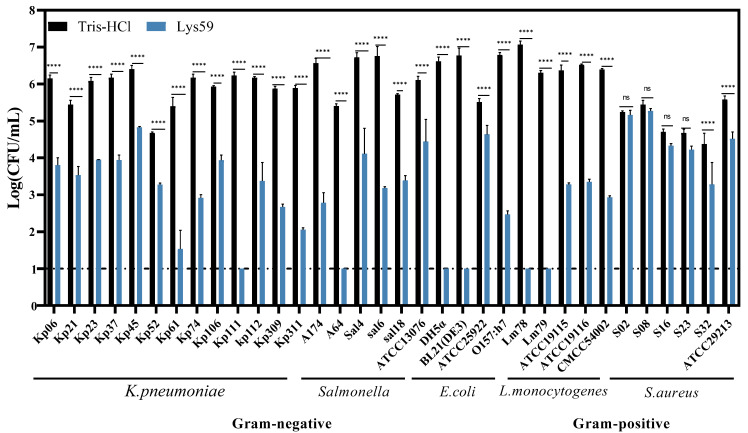
The bactericidal spectrum of Lys59. The data are presented as the means ± standard deviations of three replicates. Statistical significance was calculated using two-way ANOVA followed by Tukey’s multiple comparison test. **** *p* < 0.0001; ns, not significant.

A total of 34 bacterial strains, encompassing both Gram-positive and Gram-negative species, were used to investigate the lytic spectrum of Lys59. The result was shown in Figure 4. Lys59 exhibited bactericidal activity against all tested Gram-negative bacteria, including *K. pneumoniae*, *Salmonella enterica*, and *Escherichia coli*. Interestingly, some Gram-positive bacterial strains, such as *L. monocytogenes* and *S. aureus* were also degraded by Lys59. Notably, its bactericidal activity against *L. monocytogenes* 78 and *L. monocytogenes* 79 reduced viable cell counts to undetectable levels. Therefore, Lys59 is a broad-spectrum endolysin.

### 3.4. Effect of pH, Temperature, NaCl, and Metal Ions on Antibacterial Activity

**Figure 5:**
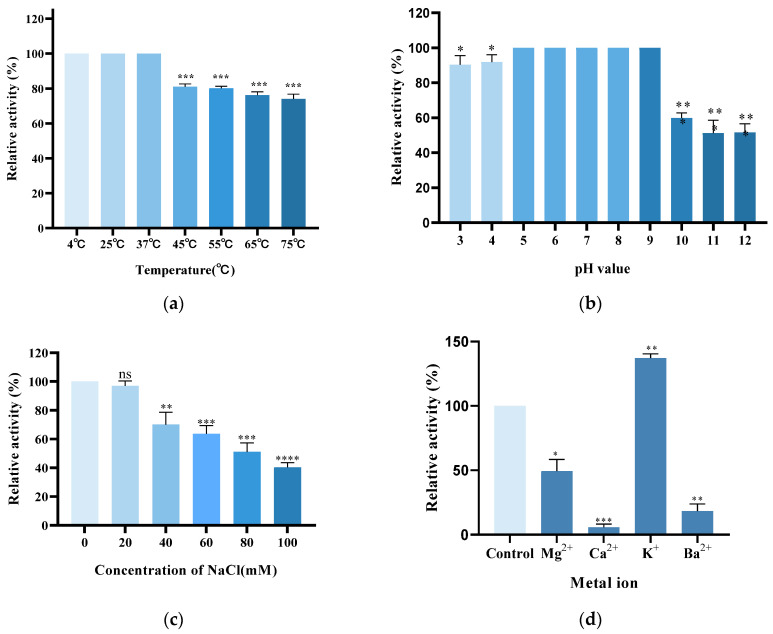
Biochemical characterization of the lytic activity of purified Lys59. The effect of Temperature (**a**), pH (**b**), salt concentration (**c**) and metal ions (**d**) on the lytic activities of Lys59 against *K. pneumoniae* 61. The data are presented as the means ± standard deviations of three replicates. Statistical significance was calculated using two-way ANOVA followed by Tukey’s multiple comparison test. * *p* < 0.05; ** *p* < 0.01; *** *p* < 0.001; **** *p* < 0.0001; ns, not significant.

The bactericidal activity of Lys59 was evaluated under different conditions such as temperatures (4−75 °C), pH (3−12) and NaCl concentrations (0−100 mM). Lys59 exhibited stable and high activity across temperatures ranging from 4 °C to 37 °C. Lys59 exhibited 80% activity at 55 °C and retained 76% and 74% at 65 °C and 75 °C, respectively (Figure 5a). The bactericidal activity was the highest and relatively stable under pH 5 to pH 9. However, under higher pH conditions (pH > 10.0), this activity dropped below 60% (Figure 5b). Notably, 90% of the lytic activity was still retained at pH 3−4. To assess how salinity affects Lys59, its lytic activity was evaluated across varying concentrations of NaCl. The results showed that the lytic activity of Lys59 decreased significantly as the salt concentration increased from 0 mM to 100 mM (Figure 5c). Lys59 endolysin had the highest lytic activity between 0 and 20 mM NaCl. Above 60% of the activity was retained at NaCl concentrations between 40 and 60 mM. The activity of Lys59 dropped below 40% when the concentration exceeded 80 mM. Therefore, NaCl can significantly affect the antibacterial activity of Lys59.

To evaluate the influence of metal ions, lytic activity of Lys59 was measured with or without various divalent cations (Mg^2+^, Ca^2+^, Ba^2+^, and K^+^; 1 mM). Compared to the control, the addition of KCl enhanced the activity of Lys59 to 137%. In contrast, the other metal ions significantly inhibited its activity (Figure 5d). Notably, Ca^2+^ reduced the antibacterial activity of Lys59 to below 10%, whereas Mg^2+^ and Ba^2+^ decreased it to 50% and 18%, respectively.

### 3.5. Metal Ions Binding Sites of Lys59

**Figure 6:**
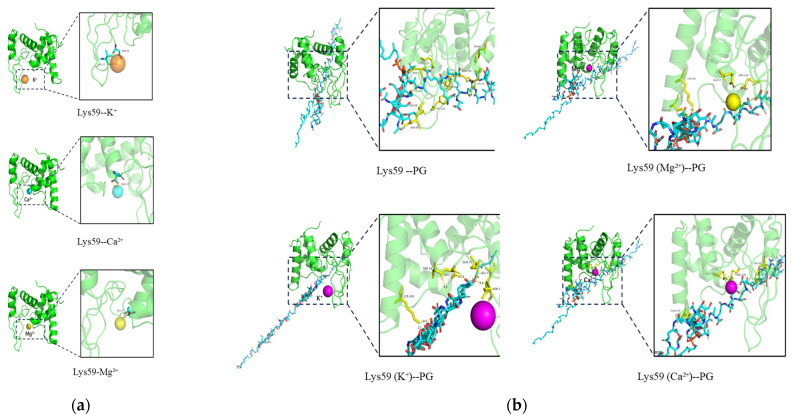
Molecular docking analysis of Lys59. (**a**) Binding sites of metal ions on the Lys59 protein. (Lys59-K^+^, ipTM = 0.87, pTM = 0.93; Lys59-Mg^2+^, ipTM = 0.89, pTM = 0.93; Lys59-Ca^2+^, ipTM = 0.86, pTM = 0.92). (**b**) Docking interactions between Lys59 and PG. (The average docking scores were −387.40 for Lys59–PG, −230.08 for Lys59 (K^+^)–PG, −192.30 for Lys59 (Mg^2+^)–PG, and −172.85 for Lys59 (Ca^2+^)–PG).

All the metal ions were docked with Lys59 protein except Ba^2+^ (Ba^2+^ is not present in the Alphafold3 metal ion ligand library). Docking results showed that Ca^2+^ and Mg^2+^ bind to GLU17, whereas K^+^ interacts with ASN35 and VAL57 (Figure 6a). Both ipTM and pTM exceed 0.85, indicating a highly reliable structure. This structure was further docked with Peptidoglycan (PG) and showed strong interactions (Figure 6b). In all docking results, interactions were observed between peptidoglycan and the GLU17 residue of Lys59. However, Ca^2+^ and Mg^2+^ coordinate with the GLU17 residue, thereby interfering with peptidoglycan binding. In contrast, K^+^ forms a more complementary and stable catalytic pocket through interactions with SER16, GLU17, and ASN35.

### 3.6. Eradication of K. pneumoniae Biofilm by Lys59

**Figure 7:**
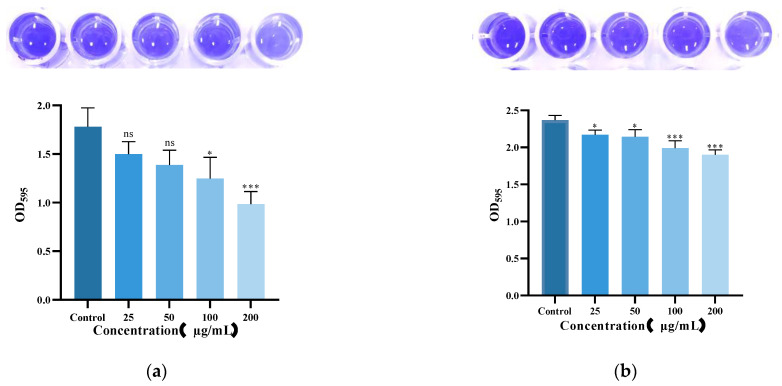
Anti-biofilm activity of Lys59 against *K. pneumoniae* 61. (**a**) Disruption of biofilms measured by crystal violet staining. (**b**) Inhibition of biofilm formation. The data are presented as the means ± standard deviations of three replicates. Statistical significance was calculated using two-way ANOVA followed by Tukey’s multiple comparison test. * *p* < 0.05; *** *p* < 0.001.

To evaluate the antibiofilm activity of Lys59, biofilm of *K. pneumoniae* 61 was grown at 37 °C for 48 h and then treated with Lys59. The result was shown in Figure 7. Lys59 removed the biofilms in a concentration-dependent manner. Treatment with 200 µg/mL of Lys59 reduced the biofilm mass by 44.6% compared to the control (Figure 7a). On this basis, we further evaluated the inhibitory effect of Lys59 on *K. pneumoniae* 61 biofilm formation at various concentrations. The results showed that Lys59 exhibited limited antibiofilm activity. Even at the highest concentration (200 µg/mL), the biofilm mass was reduced by only 19.7% compared to the untreated control (Figure 7b).

## 4. Discussion

The emergence and dissemination of multidrug-resistant pathogens have posed formidable challenges to clinical therapy. Endolysins exhibit rapid bactericidal activity and a low propensity for resistance development, indicating their potential as promising biological agents for pathogen control. Among the reported phage endolysins, those exhibiting high bactericidal activity against *K. pneumoniae* mainly originate from phages of other genera, such as the endolysin LysSS from *Salmonella* phage SS3e [21]. In contrast, endolysins derived from *K. pneumoniae* phages generally display slow kinetics or weak antibacterial activity as shown by LysG24 from *K. pneumoniae* phage vB_KpnS_MK54 [22]. Here, we cloned and expressed the endolysin Lys59 from a *K. pneumoniae* phage with potent antibacterial activity (ORF59, annotated as lysozyme).We further investigated its bactericidal activity against Gram-positive and Gram-negative bacteria, stability, metal ion regulatory effect, and biofilm eradication capacity.

The protein, named Lys59, was successfully overexpressed in *E. coli.* NCBI CD-Search domain prediction identified a conserved lambda-lys domain, a hallmark of lytic transglycosylases that hydrolyze the β-1,4-glycosidic bond between N-acetylmuramic acid (MurNAc) and N-acetylglucosamine (GlcNAc) within the peptidoglycan backbone [23]. Further analysis indicated that Lys59 possesses thirteen sugar-binding sites and a catalytic residue (GLU17). The former are presumed to anchor the enzyme to the bacterial peptidoglycan layer, whereas the latter is essential for the bacteriolytic activity of the endolysin [24]. Lys59 carries a positive charge at N-terminus and contains an amphipathic α-helix at C-terminus. The positively charged N-terminus facilitates initial electrostatic attraction to the negatively charged bacterial surface, while the cationic region of the C-terminal amphipathic helix interacts with anionic phosphate groups of lipid A present in the outer membrane of bacteria, promoting stable contact of the endolysin to target bacteria. Subsequently, the hydrophobic part of the amphipathic helix may induce membrane penetration of the endolysin [25]. The endolysins LNT113 and LNT103 possess an amphipathic α-helix structure and can effectively lyse *E. coli* and *A. baumannii* [26,27]. This may be the reason that Lys59 can penetrate the outer membrane of Gram-negative bacteria and kill *K. pneumoniae*.

To further evaluate the bactericidal activity of Lys59, the protein was purified using a nickel column. Lys59 exhibits strong lytic activity against *K. pneumoniae* in a dose-dependent manner. Lys59 still exhibits high lytic activity at 50 µg/mL and reduces the viable count of *K. pneumoniae* 61 by approximately 4.0 log. This reduction is greater than the 1.9 log decrease achieved by the *K. pneumoniae* phage-derived endolysin Lys41 (90 µg/mL) against *K. pneumoniae* [28]. Similarly, the *E. coli* phage-derived endolysin EC340 at 40 µg/mL achieved approximately a 1.0 log reduction against *K. pneumoniae* [29]. However, the antibacterial activity in this study was assessed in a buffer condition of 20 mM Tris-HCl. Vázquez [30] et al. demonstrated that the bactericidal effect of phage endolysins is highly dependent on the tonicity and ionic strength of the buffer used in the experiment. Therefore, the antibacterial activity reported in this study has certain limitations. Future studies should further investigate its antibacterial activity under physiological conditions to facilitate further development and application. Lys59 exhibits bactericidal activity against all tested Gram-negative bacteria. Furthermore, Lys59 is also capable of lysing Gram-positive bacteria, such as *L. monocytogenes* and *S. aureus*. Notably, its broad-spectrum activity surpasses that of previously reported endolysins in Gram-negative bacteriophages. LysSP1 derived from *Salmonella* phages effectively kills multiple Gram-negative bacteria but is ineffective against Gram-positive bacteria [31]. The lytic activity of Lysqdvp001 against *V. parahaemolyticus* was enhanced by modifying its C-terminus with a cationic peptide [32]. The cloned Lys59 exhibited lytic activity against both Gram-negative and Gram-positive bacteria, indicating its promising potential for further development and application.

Based on various stability tests, the endolysin Lys59 was found to be highly stable under high temperatures, low salt concentrations, and a broad pH range (pH 5−9), which suggests that Lys59 is an acidophilic endolysin. Its pH-dependent activity differs significantly from that of alkaliphilic endolysins, such as LysSTG2 [33]. However, 100 mM NaCl severely inhibited the bactericidal activity of Lys59, which may affect its further development and application. In the future, encapsulation (e.g., liposomes or nanoparticles) could be considered to further explore ways to reduce the impact of physiological conditions on its bactericidal activity. Furthermore, the bactericidal activity of Lys59 was strongly inhibited by Mg^2+^, Ca^2+^ and Ba^2+^. In contrast, the addition of K^+^ significantly enhanced its lytic activity. LysSE24 from *Salmonella* phage also showed metal ion sensitivity, with its activity being suppressed by Ca^2+^ and Mg^2+^ at concentrations above 10 mM [10]. The different impact of metal ions on endolysin activity is likely attributable to their binding to distinct amino acid residues within metal ion-binding domains [34]. Our results revealed that Mg^2+^ and Ca^2+^ can bind to the catalytic residue GLU17 of Lys59. Evrard et al. proposed that the GLU residue is essential for the catalytic cleavage activity of endolysins containing a Lambda-like domain [24]. Hence, the inhibitory effect of divalent cations can be attributed to their coordination with GLU17, which directly hampers its function as a general acid catalyst residue. In contrast, K^+^ interacts with the ASN35 and VAL57 residues. Nawaz et al. have noted that some endolysins utilize a serine (SER) residue to position the catalytic water molecule [35]. Furthermore, the addition of K^+^ induces an interaction between PG and the SER16 residue. Therefore, the enhancing effect of K^+^ may be mediated by the induction of a conformational change, which enables the entire active pocket (SER16, GLU17, ASN35) to form a more complementary and stable interface with the PG substrate, thereby enhancing bactericidal efficacy. Biofilm is crucial for survival and transmission of *K. pneumoniae*. Phage-derived enzymes have been considered as an effective strategy to inhibit and eradicate biofilm of pathogens. the potential role of Lys59 in eliminating mature biofilms was investigated. In this study, 44.6% of *K. pneumoniae* mature biofilms was removed after treatment with 200 µg/mL Lys59.

Overall, these data identify Lys59 as a promising candidate for developing effective agents against *K. pneumoniae* infections. However, the overall safety and application potential of Lys59 require further extensive investigation.

## 5. Conclusions

In summary, the endolysin Lys59 encoded by phage VB_KpP_HS106 of *K. pneumoniae* was successfully cloned and expressed. It possesses an amphipathic helix at the C-terminus, the catalytic residue Glu17, and peptidoglycan-binding sites, all of which are critical for its bactericidal activity. Lys59 at 50 µg/mL reduces the viable count by approximately 4.0 log. Lys59 shows broad lytic activity, high stability, and antibiofilm capacity. Lys59 displays activity not only against *K. pneumoniae* but also toward a wide range of both Gram-negative and Gram-positive bacteria. The bactericidal activity of Lys59 was significantly enhanced when combined with K^+^. Lys59 can serve as a promising alternative to antibiotics for combating *K. pneumoniae*.

## Figures and Tables

**Table 1:** The strains used in this study.

**Gram-Negative Bacteria**			
**Strains**	**Name**	**Drug Resistance**	**Source**
*Klebsiella pneumoniae*	06	AMP, CFZ, CTX, DOX, COL	cattle barn soil
*Klebsiella pneumoniae*	21	AMP, CFZ, CTX, CAZ, DOX, COL	cattle bedding
*Klebsiella pneumoniae*	23	AMP, CFZ, CTX, CAZ, IPM, DOX, SMZ, COL	cattle bedding
*Klebsiella pneumoniae*	37	AMP, CFZ, CTX, TET, GEN, CHL, SMZ, COL	raw milk (cow)
*Klebsiella pneumoniae*	45	AMP, CFZ, CAZ, VAN, CHL, SMZ, COL	raw milk (cow)
*Klebsiella pneumoniae*	52	AMP, CFZ, CTX, CAZ, CCL, CRO, DOX, SMZ	raw milk (cow)
*Klebsiella pneumoniae*	61	AMP, CFZ, CCL, CTX, CAZ, CRO, IMP, TET, DOX, GEN, CHL, SMZ, COL	raw milk (cow)
*Klebsiella pneumoniae*	74	AMP, CFZ, CTX, CAZ, CCL, CRO, SMZ, GEN, DOX, TET	raw milk (cow)
*Klebsiella pneumoniae*	106	AMP, CFZ, CTX, CAZ, CCL, CRO, TET, CHL, SMZ, COL	raw milk (cow)
*Klebsiella pneumoniae*	111	AMP, CFZ, CTX, CRO, CCL, TET, VAN, CHL, COL	raw milk (cow)
*Klebsiella pneumoniae*	112	AMP, CFZ, CTX, CRO, CAZ, CCL, DOX, GEN, CHL, SMZ, COL	raw milk (cow)
*Klebsiella pneumoniae*	309	AMP, CFZ, CTX, CRO, CCL, TET, DOX, GEN, CHL, SMZ, COL	raw milk (cow)
*Klebsiella pneumoniae*	311	AMP, CFZ, CTX, CAZ, CRO, CCL, TET, DOX, GEN, VAN, CHL, SMZ, COL	raw milk (cow)
*Salmonella enterica*	A174	AMP, TET, DOX, SMZ, ERY	Customs
*Salmonella enterica*	A64	AMP, TET, DOX, CIP, OFX, SMZ, ERY, AZM, KAN, GEN	Customs
*Salmonella enterica*	Sa14	AMP, ERY	Customs
*Salmonella enterica*	Sa16	AMP, TET, CHL, SMZ, ERY	Customs
*Salmonella enterica*	Sa118	AMP, DOX, SMZ, ERY	Customs
*Salmonella enterica*	ATCC13076	ERY	ATCC
*Escherichia coli*	ATCC25922	-	ATCC
*Escherichia coli*	DH5α	-	laboratory model strain
*Escherichia coli*	BL21(DE3)	-	laboratory model strain
**Gram-Positive Bacteria**			
**Strains**	**Name**	**Drug Resistance**	**Source**
*Listeria monocytogenes*	78	-	raw milk (cow)
*Listeria monocytogenes*	79	-	raw milk (cow)
*Listeria monocytogenes*	ATCC19115	-	ATCC
*Listeria monocytogenes*	ATCC19116	-	ATCC
*Listeria monocytogenes*	CMCC54002	-	CMCC
*Staphylococcus aureus*	02	AMP, CZO, SMZ	Nipple milk
*Staphylococcus aureus*	08	AMP, CZO, TET, DOX, GEN	Nipple milk
*Staphylococcus aureus*	16	AMP	Nipple milk
*Staphylococcus aureus*	23	AMP, CZO, TET, DOX, GEN	Nipple milk
*Staphylococcus aureus*	32	AMP	Nipple milk
*Staphylococcus aureus*	ATCC29213	-	ATCC

## Data Availability

The original contributions presented in this study are included in the article/Supplementary Material. Further inquiries can be directed to the corresponding author.

## Supplementary Materials

The following supporting information can be downloaded at: https://www.mdpi.com/article/10.3390/microorganisms14051027/s1, Table S1: Primers used in this experiment; Table S2: Physicochemical Properties of Lys59; Figure S1: Hydrophobicity analysis of Lys59; Figure S2: Presence of secondary structures at various positions in the Lys59 amino acid sequence; Figure S3: Prediction of the amphipathic α-helix in Lys59.
