# Supplementary material for: A New Endolysin Lys59: A Broad-Spectrum Phage Endolysin Targeting Both Gram-Negative and Gram-Positive Bacteria
Source: Microorganisms. 2026 Apr 30;14(5):1027. doi: 10.3390/microorganisms14051027 (PMC13209667; doi:10.3390/microorganisms14051027)
Supplement: Supplementary file 1 [file microorganisms-14-01027-s001.zip › microorganisms-4258481-supplementary.pdf]

Supplementary Table S1

Primers used in this experiment.

Name	Sequence
Lys59-EcoRI-F	GGAATTCATGATTAGTAAGCAAGTAT
Lys59--XhoI-R	CCCTCGAGTGCTACTTTGCCTCCGAATT

Supplementary Table S2

Physicochemical Properties of Lys59.

Index	Lys59
Theoretical pI	9.58
Total number of negatively charged residues (Asp + Glu)	13
Total number of positively charged residues (Arg + Lys)	23
Instability index	35.19(stable)
Aliphatic index	86.94
Grand average of hydropathicity (GRAVY)	-0.192
Alpha helix content	43.95%
β-sheet content	2.55 %
Random coil content	53.50 %

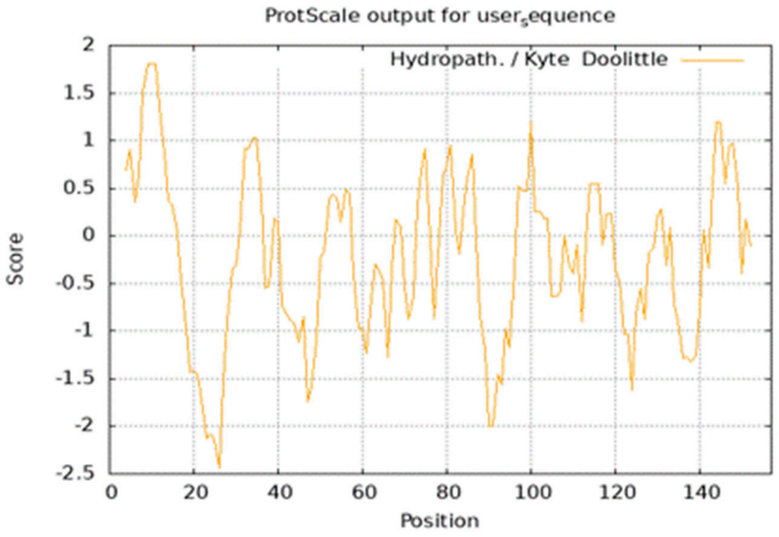

Supplementary Figure S1. Hydrophobicity analysis of Lys59.

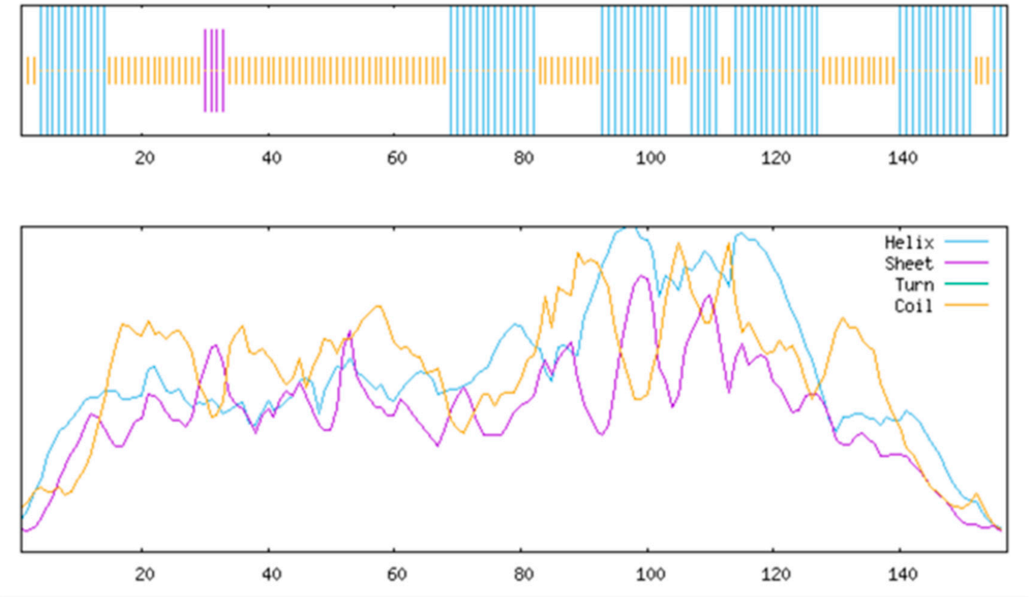

Supplementary Figure S2. Presence of secondary structures at various positions in the Lys59 amino acid sequence.

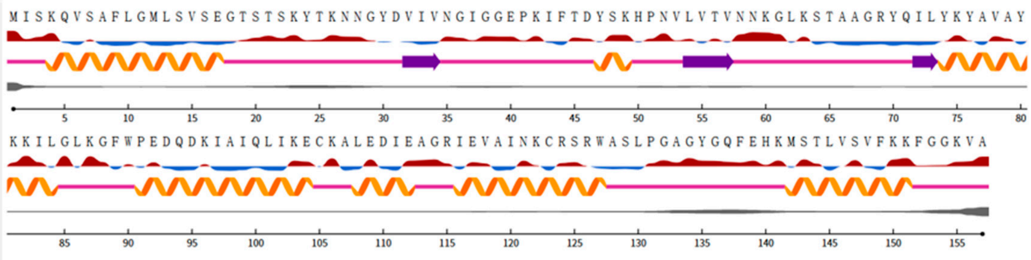

Supplementary Figure S3. Prediction of the amphipathic α-helix in Lys59.
